# Dimorphic male scutal patterns and upper-eye facets of *Simulium mirum* n. sp. (Diptera: Simuliidae) from Malaysia

**DOI:** 10.1186/s13071-016-1393-9

**Published:** 2016-03-09

**Authors:** Hiroyuki Takaoka, Van Lun Low, Mohd Sofian-Azirun, Yasushi Otsuka, Zubaidah Ya’cob, Chee Dhang Chen, Koon Weng Lau, Maria Lourdes Lardizabal

**Affiliations:** Institute of Biological Sciences, Faculty of Science, University of Malaya, Kuala Lumpur, Malaysia; Research Center for the Pacific Islands, Kagoshima University, Korimoto 1-21-24, Kagoshima, Japan; International Tropical Forestry Programme, Faculty of Science and Natural Resources, Universiti Malaysia Sabah, Jalan UMS, Kota Kinabalu, Sabah Malaysia

**Keywords:** *Simulium*, New species, Male dimorphism, Sabah, Sarawak

## Abstract

**Background:**

A species of *Simulium* in the *Simulium melanopus* species-group of the subgenus *Simulium* (formerly misidentified as *S. laterale* Edwards from Sabah and Sarawak, Malaysia) is suspected to have dimorphic male scutal color patterns linked with different numbers of upper-eye facets. This study aimed to confirm whether or not these two forms of adult males represent a single species.

**Methods:**

DNA sequences generated from four genetic loci, the mitochondrial-encoded COI, COII, 12S rRNA and 16S rRNA genes, of both forms of *Simulium* sp. males were compared with each other and also with those of the females and larvae of the same species. Four other related *Simulium* spp. were also used for comparison.

**Results:**

Both the concatenated dataset and single-locus phylogenetic analyses indicate that the two distinct morphological males of *Simulium* sp. are indeed conspecific, and represent, together with their associated females and larvae, a distinct species.

**Conclusions:**

Based on DNA analyses, *Simulium* sp. is proven to show dimorphism in males and is herein described as a new species, *Simulium mirum* Takaoka, Sofian-Azirun & Low. This is the first report of such a novel species among the family Simuliidae.

**Electronic supplementary material:**

The online version of this article (doi:10.1186/s13071-016-1393-9) contains supplementary material, which is available to authorized users.

## Background

The black flies (Diptera: Simuliidae) in Sabah and Sarawak, Malaysia, in the northern part of Borneo, were recently reviewed [1–3]. So far, 38 species have been recorded from Sabah and Sarawak, all of which are classified in three subgenera of the genus *Simulium*: 18 species in *Gomphostilbia*, three species in *Nevermannia* and 17 species in *Simulium* [3–7]. Biting habits of females and other biological aspects of these East Malaysian black fly species remain unknown, except for one female of *S. nigripilosum* Edwards, which was caught by a hand net while flying around a human along the trail to the peak of Mt. Kinabalu, 2 km up from the Carson Waterfall, on September 10, 2007.

In a revision of eight species of the *Simulium melanopus* species-group of the subgenus *Simulium* from Sabah, one species that was misidentified as *Simulium laterale* Edwards by Smart & Clifford [8], has been treated as *Simulium* sp. (*sensu* Smart & Clifford, 1969) [3]. This species is herein described as new.

**Fig. 1:**
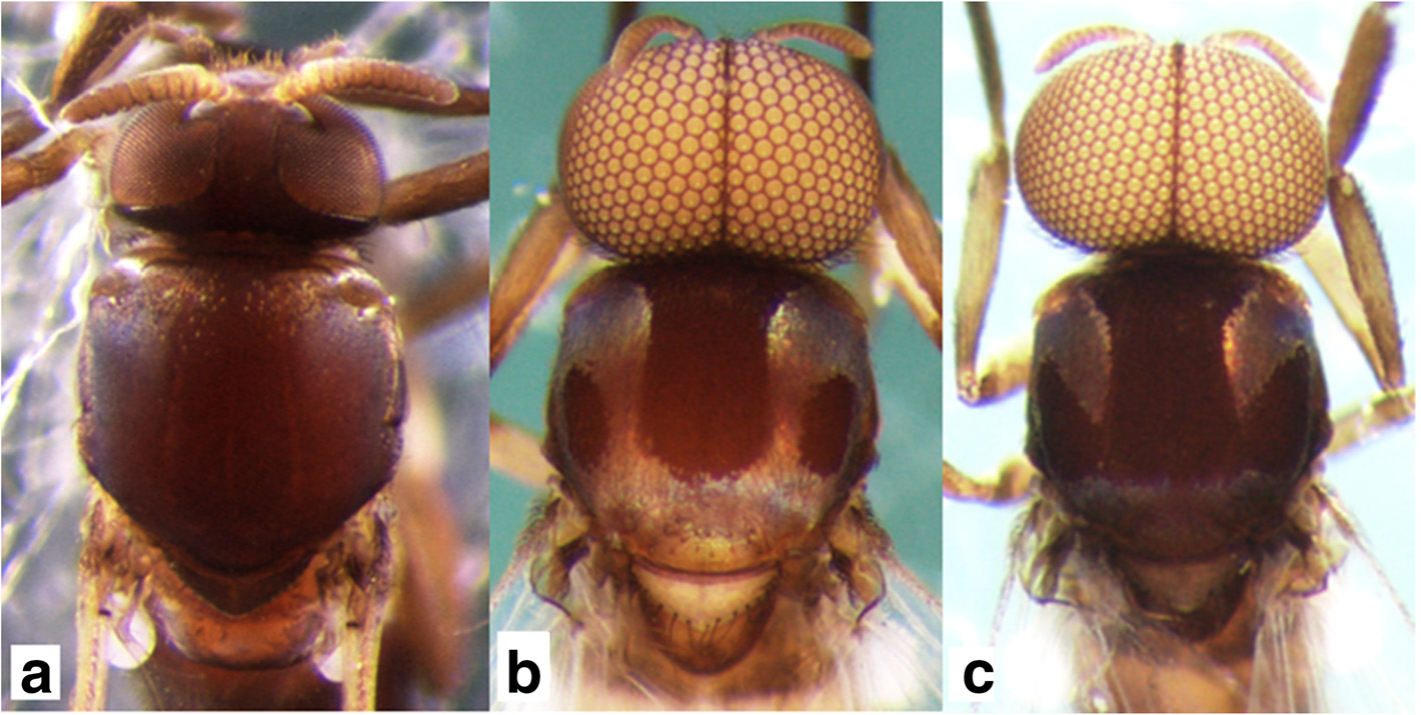
Heads and thoraces of the female, and two male forms of *Simulium mirum* n. sp. (**a**) Female. **b** Form A male. **c** Form B male. All are shown in dorsal view

This new species was suspected of being dimorphic in the males. Form A males have a white pruinose scutum with a medial vitta and lateral, round, nonpruinose spots (Fig. 1b) and upper-eye facets in 12–14 vertical columns and 15 or 16 horizontal rows. Form B males have a white pruinose scutum with an inverted-T-shaped, large, medial, nonpruinose portion (Fig. 1c) and upper-eye facets in 16 or 17 vertical columns and 17 or 18 horizontal rows [3]. To test this hypothesis, we adopted four genetic markers most commonly used for black fly phylogeny and systematics [9–11]. Genetic evidence that supports the conspecificity of the two forms of the males of this new species is provided, and a description of *Simulium* sp. as a new species is given based on females, males, pupae and larvae.

## Methods

### Taxa used for DNA analyses

To determine whether form A and form B males of *Simulium* sp. comprised one species, males of both forms together with females (all reared from pupae) and larvae collected from Mt. Kinabalu, Sabah, and Bakalalan, Sarawak, Malaysia, were used for DNA analyses. Other related species used for comparison were *S. crassimanum* Edwards, *S. laterale*, *S. nigripilosum* and *S. maklarini* Takaoka, all Sabah members of the *S. melanopus* species-group [3]. For 16S rRNA, sequences of *S. bishopi* Takaoka & Davies (AB093105) from Peninsular Malaysia, *S. dumogaense* Takaoka & Roberts (AB093106) and *S. tumpaense* Takaoka & Roberts (AB093110) from Indonesia, *S. melanopus* Edwards (AB093108) and *S. taalense* Takaoka (AB093109) from the Philippines available from the NCBI GenBank database were also included in phylogenetic analyses.

### DNA isolation, polymerase chain reaction (PCR) and DNA sequencing

The genomic DNA was isolated from each individual, using the i-genomic CTB DNA Extraction Mini Kit (iNtRON Biotechnology Inc., Seongnam, South Korea). The DNA amplifications by PCR were conducted using an Applied Biosystems Veriti 96-Well Thermal Cycler (Applied Biosystems Inc., Foster City, CA, USA). Amplifications of the mitochondrial-encoded COI, COII, 12S rRNA and 16S rRNA genes were performed in a reaction mixture containing 50–100 ng of genomic DNA, 25 μL of NEXpro e PCR 2x Master Mix (Genes Labs Inc., Gyeonggi-do, South Korea), and 10 pmol of each forward and reverse primer. The primers used in this study were adopted from Folmer et al. [12] for COI, Simon et al. [13] for COII, Kocher et al. [14] and Simon et al. [13] for 12S rRNA, and Xiong & Kocher [15] for 16S rRNA. The PCR products were sequenced in both directions using the Big Dye Terminator v3.1 kit and run on an ABI 3730XL Genetic Analyzer (Applied Biosystems Inc.).

### DNA sequence analyses

Sequences were assembled and edited using ChromasPro Version 1.7.6 (Technelysium Pty Ltd., Australia). All sequences were preliminarily aligned using CLUSTAL X and edited using BioEdit 7.0.9.0 [16]. The COI, COII, 12S rRNA and 16S rRNA sequences generated in this study were deposited in the NCBI GenBank database under accession numbers KT207386-KT207457.

Congruence between separate genes was tested using the partition homogeneity test [17] implemented in PAUP 4.0b10 [18]. The results showed no significant differences among separate gene regions (*P* = 0.459); hence, data were concatenated for further analyses. The aligned sequences of single genes and the concatenated dataset were subjected to Bayesian inference (BI) analysis using four chains of Markov chain Monte Carlo (MCMC) implemented in MrBayes 3.1.2 [19]. Neighbour-joining (NJ) and maximum parsimony (MP) analyses were performed using PAUP 4.0b10. The MP tree was constructed using the heuristic search option, 100 random sequences additions, tree bisection reconnection (TBR) branch swapping, and unordered and unweighted characters. The NJ tree was estimated using Kimura’s two-parameter model of substitution (K2P distance) evolution model. Maximum likelihood (ML) analysis was performed with GTR substitution model using PhyML 3.0 [20]. In this study, *S.* (*Simulium*) *nobile* de Meijere and *S.* (*Gomphostilbia*) *leparense* Takaoka, Sofian-Azirun & Ya’cob were chosen as outgroups for the construction of phylogenetic trees. Uncorrected (p) pairwise genetic distances among species were estimated using PAUP 4.0B10.

### Description of a new species

The methods of description and illustration, and terms for morphological features used here, follow those of Takaoka [21]. Specimens used for the description are listed in Type Material.

## Results

### DNA analyses

**Fig. 2:**
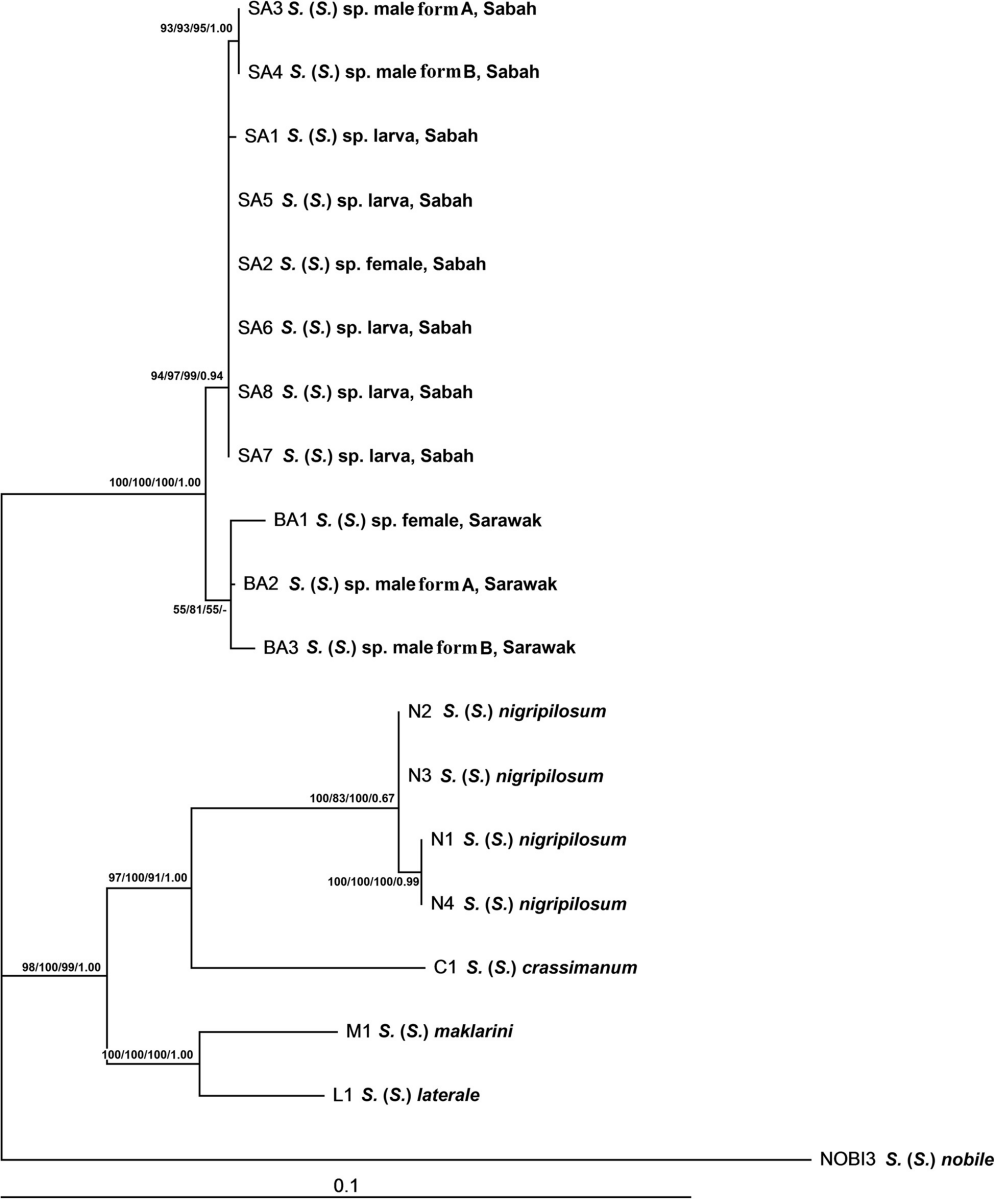
Maximum likelihood phylogenetic tree of the *Simulium melanopus* species-group from East Malaysia based on concatenated sequences of COI, COII, 12S rRNA and 16S rRNA genes. Bootstrap and posterior probability values [ML/MP/NJ/BI] are shown on the branches

The concatenated COI + COII + 12S rRNA + 16S rRNA phylogenetic tree revealed two main genetic clades. One well-supported clade comprised *Simulium* sp. from Sabah and Sarawak, with its respective sub-clades according to populations. The other well-supported clade comprised other members of the *S. melanopus* species-group in Malaysia: *S. crassimanum*, *S. laterale*, *S. nigripilosum* and *S. maklarini* (Fig. 2)*.*

Both the concatenated dataset (Fig. 2) and single-locus phylogenetic analyses (Additional file 1: Figures S1–S4) indicate that form A and form B males of *Simulium* sp. are conspecific, and both forms of males are associated with females and larvae of *Simulium* sp. The intraspecific genetic distance of *Simulium* sp. ranged from 0 to 1.10 %. *Simulium* sp. was distinctly separated from four closely related taxa; the interspecific genetic distance between species pairs was as follows: *Simulium* sp. *vsS. crassimanum* (5.80–6.03 %), *Simulium* sp. *vsS. laterale* (5.34–5.71 %), *Simulium* sp. *vsS. nigripilosum* (5.75–6.26 %) and *Simulium* sp. *vsS. maklarini*, (5.89–6.26 %) (Table 1).

**Table 1:** Ranges of intra- and inter-specific pairwise genetic distances based on concatenated gene sequences of COI (630 bp), COII (614 bp), 12S rRNA (434 bp) and 16S rRNA (508 bp) genes

	1	2	3	4	5
1. *S. mirum* n. sp.	0.00–1.10				
2. *S. crassimanum*	5.80–6.03	N/A			
3. *S. laterale*	5.34–5.71	5.16	N/A		
4. *S. maklarini*	5.89–6.26	5.89	3.01	N/A	
5. *S. nigripilosum*	5.75–6.26	4.52–4.66	4.93–5.02	5.11–5.21	0.00–0.37

### Description of new species

*Simulium mirum* Takaoka, Sofian-Azirun & Low

*Simulium* (*Simulium*) *laterale* (not Edwards): Smart & Clifford [8], pages 22–26 (Female,

male, pupa and larva)

*Simulium* (*Simulium*) sp.: Takaoka et al. [3], page 28.

### Female (*n* = 10)

**Fig. 3:**
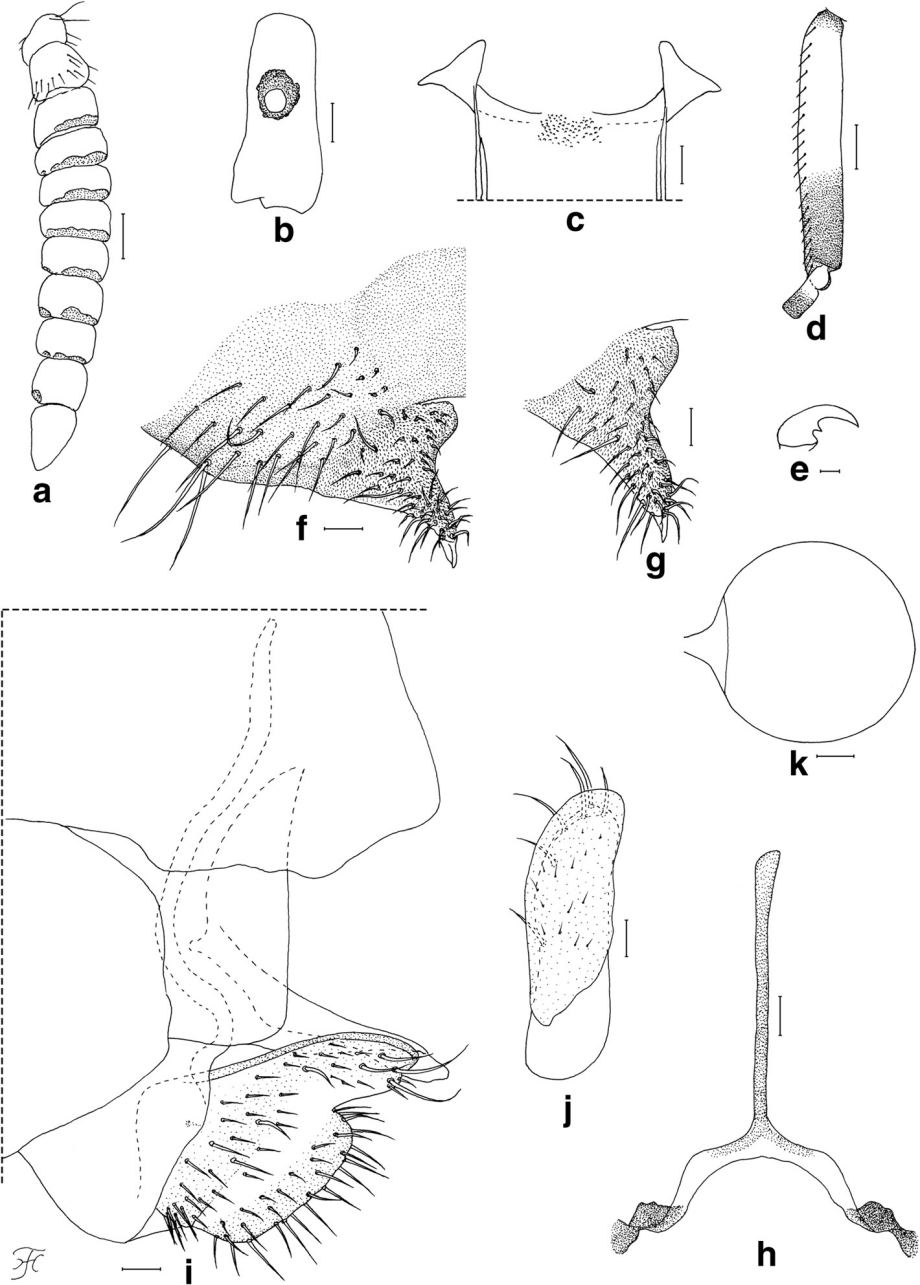
Female of *Simulium mirum* n. sp. (**a**) Antenna (left side; outer view). **b** Third segment of maxillary palp with sensory vesicle (left side; frontal view). **c** Cibarium (frontal view). **d** Basitarsus and second tarsomere of hind leg (left side; outer view). **e** Claw. **f** Eighth sternite and ovipositor valve (right half; ventral view). **g** Ovipositor valve (right side; anteroventral view). **h** genital fork (ventral view). **i** Terminalia showing eighth sternite, ovipositor valve, paraproct and cercus (lateral view). **j** Paraproct showing anteroventral sclerotized plate (left side; anterior view). **k** Spermatheca. Scale-bars: 0.1 mm for (**d**); 0.05 mm for (**a**); 0.02 mm for (**b**), (**c**) and (**f**)–(**k**); 0.01 mm for (**e**)

Body length 2.5–2.8 mm. *Head* (Fig. 1a). Frons black, strongly shiny when illuminated at certain angles, with several dark stout hairs along each lateral margin and several shorter hairs near lower margin on each side; frontal ratio 1.19: 1.00: 1.25; frons-head ratio 1.00:4.25. Fronto-ocular area shallow, rounded laterally. Clypeus black, whitish-gray pruinose, shiny when illuminated at certain angles, moderately covered with dark stout hairs. Labrum 0.62 times as long as clypeus. Antenna composed of scape, pedicel and nine flagellomeres, brownish black except scape, pedicel and first flagellomere dark yellow when viewed ventrally, or scape, basal one-third of pedicel dark yellow, and apical two-thirds of pedicel and first flagellomere medium brown when viewed posterodorsally; flagellomeres 1–8 each with pit-like depressions of irregular shape, bearing numerous sensilla, near apical margin on outer and inner surface (though flagellomere 8 with such depression only on outer surface) (Fig. 3a). Maxillary palp composed of five segments, proportional lengths of third, fourth, and fifth segments 1.00: 1.09: 2.23; sensory vesicle (Fig. 3b) ellipsoidal, 0.27 times length of third segment, with medium-sized or large round opening. Maxillary lacinia with 12 inner and 14 or 15 outer teeth. Mandible with 22 inner and 11 outer teeth. Cibarium (Fig. 3c) with 75 min processes medially; cornuae moderately developed. *Thorax.* Scutum black, thinly whitish-gray pruinose and brilliantly shiny, in particular, bluish iridescent on shoulder and lateral portion along each lateral margin when illuminated at certain angles, moderately covered with medium-brown short hairs interspersed with dark-brown long upright hairs on prescutellar area. Scutellum brownish-black, with dark-brown upright long and short hairs. Postnotum brownish-black, whitish-gray pruinose and shiny when illuminated at certain angles, and bare. *Legs.* Foreleg: coxa yellow; trochanter and femur dark brown to brownish black; tibia dark brown with medial portion medium brown, brilliantly iridescent widely on outer surface; tarsus black; basitarsus with thick dorsal hair crest, much inflated, 3.92 times as long as its greatest width. Midleg: coxa, femur and tibia brownish black; tibia brilliantly iridescent widely on posterior surface; tarsus dark brown except basal five-sixths of basitarsus and base of second tarsomere yellowish white. Hind leg: brownish black except trochanter dark brown, little more than basal half to basal three-fifths of basitarsus and basal half of second tarsomere yellowish white; tibia brilliantly iridescent widely on posterior surface; basitarsus (Fig. 3d) nearly parallel-sided, 6.22 times as long as its greatest width, 0.64 and 0.62 times as wide as greatest widths of hind tibia and femur, respectively; calcipala (Fig. 3d) slightly longer than width at base, 0.53 times as wide as basitarsus; pedisulcus well developed; claw (Fig. 3e) with small subbasal tooth. *Wing.* Length 2.1–2.2 mm. Costa with dark spinules and hairs. Subcosta haired except apical one-fourth bare. Basal section of vein R fully haired; R_1_ with dark spinules and hairs; R_2_ with dark hairs only. Hairs at base of radial vein dark brown. Basal cell absent. *Abdomen.* Basal scale dark brown, with fringe of dark long hairs. Dorsal surface of abdomen medium to dark brown, moderately covered with dark brown hairs; tergite 2 with pair of large brilliantly iridescent dorsolateral spots broadly connected medially to each other; tergites 6–8 shiny. Ventral surface medium brown except segment 2 ochreous; abdominal segment 7 with large sternal plate medially. *Terminalia.* Sternite 8 (Fig. 3f) slightly depressed medially, covered with 18–31 long stout and short to medium-long fine hairs on each side. Ovipositor valves (Fig. 3f, g) bent ventrally, making right angle to sternite 8, each tapered apically with transparent bare round apex, membranous except narrow area along inner margin well sclerotized, and covered with 39–46 short to long hairs; inner margins well sclerotized, moderately concave medially. Genital fork (Fig. 3h) of inverted-Y form; arms slender, each with strongly-sclerotized ridge directed dorsally. Paraproct in lateral view (Fig. 3i) much produced ventrally, covered with seven or eight medium-long to long stout hairs and about 30–36 short fine hairs on lateral surface; paraproct anteromedially with thin elongate moderately-sclerotized plate having round apical tip and 14–16 short setae scattered on its surface (Fig. 3j). Cercus in lateral view (Fig. 3i) short, with posterior margin nearly straight, 4.6 times as wide as its greatest length, and covered with numerous hairs. Spermatheca (Fig. 3k) globular, nearly as long as wide, well sclerotized except duct and area of junction with duct unsclerotized, without reticulate surface patterns; minute internal setae present.

### Male (*n* = 8 for form A, *n* = 7 for form B)

**Fig. 4:**
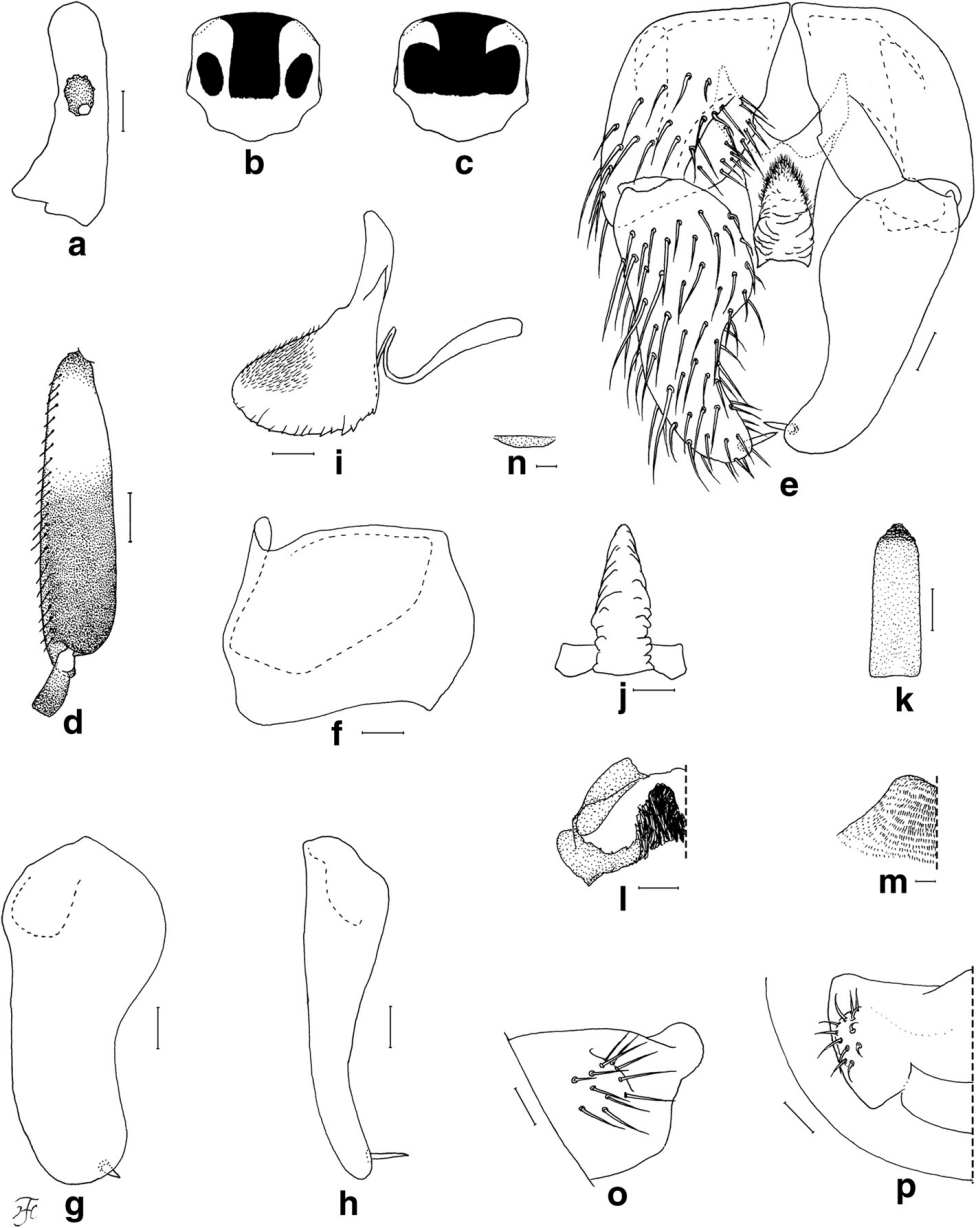
Male of *Simulium mirum* n. sp. (**a**) Third segment of maxillary palp with sensory vesicle (left side; frontal view). **b** Scutal color pattern of form A (dorsal view). **c** Scutal color pattern of form B (dorsal view). **d** Basitarsus and second tarsomere of hind leg (left side; outer view). **e** Coxites, styles and ventral plate (ventral view). **f** Coxite (right side; ventrolateral view). **g** and (**h**) Styles (right side; (**g**), ventrolateral view; (**h**), medial view). **i** Ventral plate and median sclerite (lateral view). **j** Ventral plate (caudal view). **k** Median sclerite (caudal view). **l** Paramere (right side; caudal view). **m** Aedeagal membrane (right half; caudal view). **n** Dorsal plate (ventral view). **o** and (**p**) Tenth abdominal segments and cerci (right side; (**o**), lateral view; (**p**), caudal view). Scale-bars. 0.1 mm for (**d**); 0.02 mm for (**a**) and (**e**)–(**p**)

Morphological characters are the same in form A and form B except the number of upper-eye facets and the scutal color patterns. Body length 2.5–3.5 mm. *Head* (Fig. 1b, c)*.* Nearly as wide as thorax. Upper eye consisting of large facets in 12–14 vertical columns and 15 or 16 horizontal rows on each side in form A (in 16 or 17 vertical columns and 17 or 18 horizontal rows in form B). Antenna composed of scape, pedicel and nine flagellomeres, dark-brown to brownish-black except extreme base of first flagellomere yellow; first flagellomere elongate, twice length of second one. Maxillary palp composed of five segments, proportional lengths of third, fourth, and fifth segments 1.00: 1.02: 2.33; sensory vesicle (Fig. 4a) small, ellipsoidal, 0.17–0.19 times length of third segment, and with small opening. *Thorax.* Scutum black, brilliantly iridescent with median vitta and round lateral spot on each side nonpruinose in form A (Fig. 1b and Fig. 4b) [brilliantly iridescent with inverted-T-like median portion nonpruinose in form B (Fig. 1c and Fig. 4c)], and moderately covered with dark short hairs and upright longer hairs on prescutellar area. Scutellum and postnotum as in female. *Legs.* Brownish black to black except fore coxa, basal two-thirds of mid basitarsus, little more than basal one-third of hind basitarsus (though base darkened) and basal one-third of second hind tarsomere yellow; brilliantly iridescent widely on outer surface of fore tibia and on posterolateral surface of mid tibia, and narrowly on posterior surface of base of hind tibia, when illuminated at certain angles. Fore basitarsus with thick dorsal hair crest and greatly dilated, 4.33 times as long as its greatest width. Hind basitarsus (Fig. 4d) greatly enlarged, gradually widened toward apical one-fourth, then slightly narrowed toward apex, 3.95 times as long as its greatest width, 0.93 and 1.07 times as wide as greatest widths of hind tibia and femur, respectively; calcipala nearly as long as its basal width, and 0.30 times as wide as greatest width of basitarsus; pedisulcus well developed. *Wing.* Length 2.0–2.4 mm; other features as in female except basal portion of radial vein and subcosta entirely bare. *Abdomen.* Basal scale dark brown with fringe of dark long hairs. Dorsal surface of abdomen dark brown to brownish black, moderately covered with dark hairs, with pair of brilliantly iridescent dorsolateral spots on segments 2 and 4–7, of which those on segment 2 widely connected medially to each other, and those on other segments narrowly connected anteromedially to each other; abdomen also with pair of shiny lateral portions on segments 8 and 9 when illuminated at certain angles. *Genitalia*. Coxites, styles and ventral plate in ventral view as in Fig. 4e. Coxite in ventrolateral view (Fig. 4f) nearly quadrate, 0.75 times as long as wide. Style in ventrolateral view (Fig. 4g) elongate, 1.84 times length of coxite, 2.19 times as long as its greatest width, widened from base to basal one-fourth, narrowed to little less than basal three-fifths, then nearly parallel-sided toward round apex, and with stout subapical spine; style in medial view (Fig. 4h) flattened dorsoventrally, gradually narrowed toward apex, and without basal or subbasal protuberance. Ventral plate in ventral view (Fig. 4e) with body nearly rectangular having ventrally produced median process that is bare except parts of lateral and anterior surfaces densely covered with minute setae, furnished with several teeth in vertical row on each posterolateral margin; basal arms short, stout and divergent; ventral plate in lateral view (Fig. 4i) with body and its ventrally produced process with rounded ventral apex, with dentate posterior margin, and densely covered with minute setae anteromedially and anterolaterally; ventral plate in caudal view (Fig. 4j) with body and ventrally produced median process slightly narrowed from base to basal one-fourth, widened to basal one-third, then tapered apically, bare, with several teeth in vertical row on each lateral margin. Median sclerite in lateral view (Fig. 4i) folded backward and then curved dorsally, and in posterior view (Fig. 4k) plate-like, somewhat pigmented except apicomedial portion unpigmented, nearly parallel-sided except basal portion tapered toward base. Paramere (Fig. 4l) wide basally and with several small parameral hooks apically. Aedeagal membrane (Fig. 4m) densely covered with minute setae, with short sclerotized dorsal plate (Fig. 4n). Ventral surface of tenth abdominal segment (Fig. 4o, p) without distinct hairs. Cercus (Fig. 4o, p) rounded posteriorly, with 11 or 12 distinct hairs.

### Pupa (*n* = 25)

**Fig. 5:**
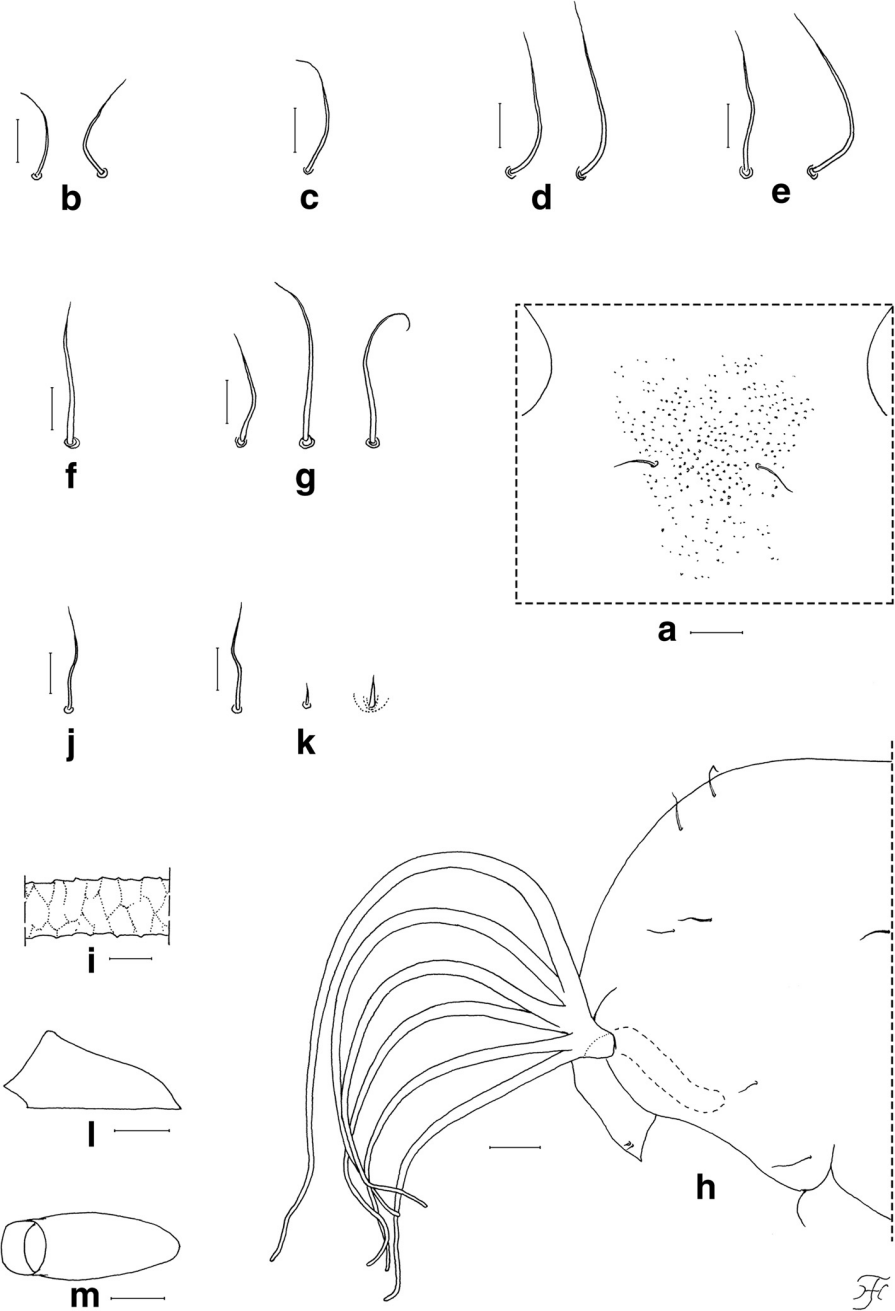
Pupa of *Simulium mirum* n. sp. (**a**) Part of face near its border to frons showing central area covered with tubercles (front view). **b** Frontal trichomes. **c** Facial trichome. **d**–**g** Thoracic trichomes (**d**, mediodorsal; **e**, anterolateral; **f**, mediolateral; **g**, ventrolateral). **h** Anterior half of thorax and gill filaments (left side; lateral view). **i** Middle part of upper filament of dorsal pair showing annular ridges forming reticulate patterns (lateral view). **j** Slender seta on dorsal surface of abdominal segment 1. **k** Short slender seta, minute slender seta and minute somewhat stout seta on dorsal surface of abdominal segment 2. **l** and (**m**) Cocoons (**l**, lateral view; **m**, dorsal view). Scale-bars: 1.0 mm for (**l**) and (**m**); 0.1 mm for (**h**); 0.05 mm for (**a**); 0.02 mm for (**b**)–(**g**) and (**i**)–(**k**)

Body length (excluding gill filaments) 2.7–3.6 mm. *Head.* Integument yellowish, bare except area surrounding facial trichomes (Fig. 5a), and each lateral surface moderately covered with minute tubercles; frons with two unbranched short trichomes (Fig. 5b) on each side; face with one unbranched short trichome (Fig. 5c) on each side; frontal trichomes shorter than facial ones. *Thorax.* Integument yellowish, bare widely on dorsal and dorsolateral surfaces of anterior half, densely covered with round tubercles on ventrolateral surface on anterior half, and sparsely or moderately covered with cone-shaped smaller tubercles on dorsal surface of posterior half; thorax with two medium-long anterodorsal trichomes (Fig. 5d), two medium-long anterolateral trichomes (Fig. 5e), one medium-long mediolateral trichome (Fig. 5f), and three ventrolateral trichomes (two medium-long, one short) (Fig. 5g), on each side; all unbranched. Gill (Fig. 5h) with six slender thread-like filaments in pairs arising from short common basal stalk; all pairs almost sessile; filaments decreasing in length and thickness from dorsal to ventral; upper filament of dorsal pair longest (1.4 mm long) and lower filament of ventral pair shortest (0.7 mm long); relative thickness of filaments from dorsal to ventral when basal portions were compared 1.00: 0.88: 0.81: 0.75: 0.69: 0.69; each filament light to medium brown, nearly parallel-sided from base to middle, then gradually tapered toward apex; upper filament of dorsal pair and lower filament of ventral pairs at angle of 90° or little more when basal portions viewed laterally; cuticular surface with marked annular ridges forming reticulate patterns (Fig. 5i), covered with minute tubercles, of which relatively larger ones on ridges and smaller ones on inter-ridge spaces. *Abdomen.* Dorsally, segment 1 yellow, other segments unpigmented; segment 1 without minute tubercles (though submedial areas moderately covered with tubercles in two pupae) and with one unbranched short slender seta (Fig. 5j) on each side; segment 2 with one unbranched short slender seta and five minute setae, of which three are stout and pigmented, two are slender and unpigmented (Fig. 5k), on each side; segments 3 and 4 each with four unbranched hooked spines and one minute slender seta on each side; segment 8 with spine-combs; segments 6–9 each with comb-like groups of minute spines in transverse row on each side; segment 9 without terminal hooks. Ventrally, all segments unpigmented; segment 5 with pair of bifid hooklets submedially and few minute setae on each side; segments 6 and 7 each with pair of bifid inner and outer hooklets and few minute setae on each side; segments 4–8 each with comb-like groups of minute spines on each side. *Cocoon* (Fig. 5l, m). Shoe-shaped, with anterior collar of moderate height, thickly woven, ochreous, not extended ventrolaterally; individual threads invisible; 3.2–3.7 mm long by 1.2–1.5 mm wide.

### Mature larva (*n* = 7)

**Fig. 6:**
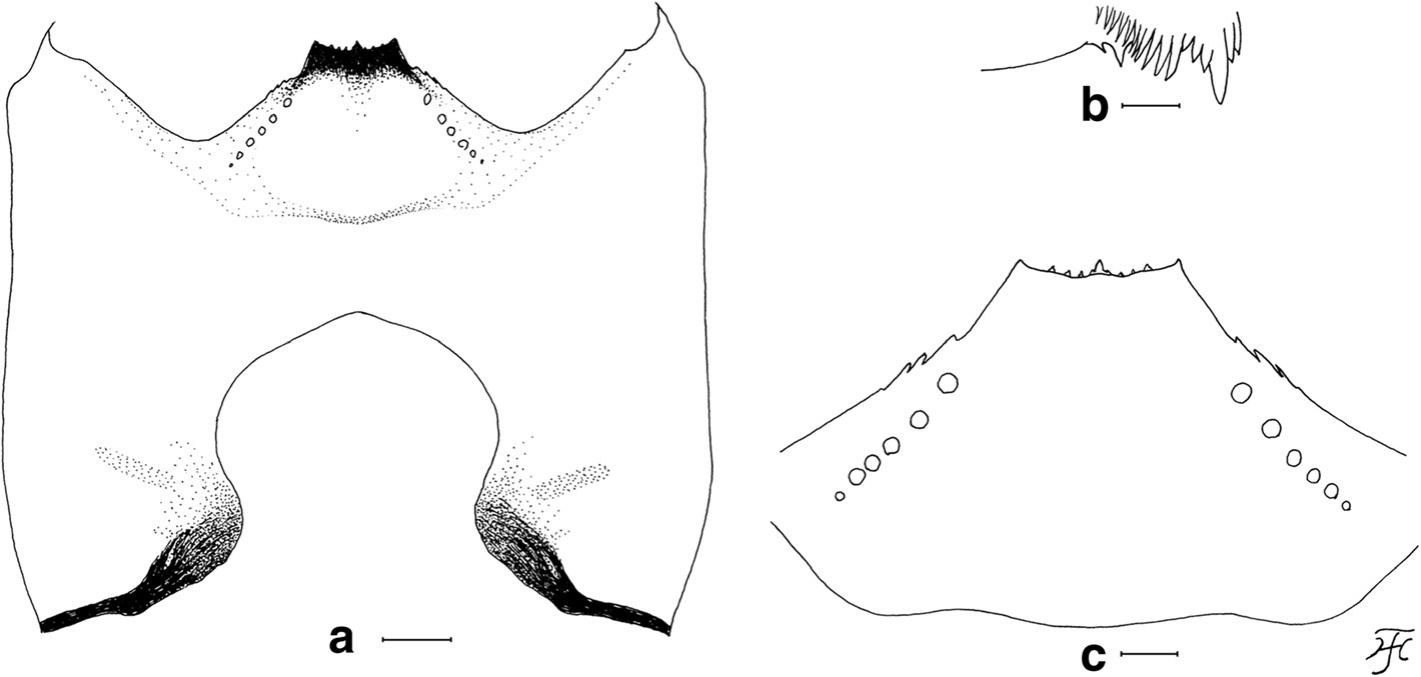
Larva of *Simulium mirum* n. sp. (**a**) Head capsule showing postgenal cleft (ventral view). **b** Mandible. **c** Hypostoma. Scale-bars: 0.05 mm for (**a**); 0.02 mm for (**b**) and (**c**)

Body length 4.8–5.2 mm. Body whitish gray with abdominal segments 6–9 faintly to moderately overlaid with ochreous to reddish brown pigments (and abdominal segments 1–5 faintly overlaid with reddish brown pigment in two larvae). Abdomen becoming slightly wider from segment 1 to segment 6, widest between segments 6 and 7. Cephalic apotome whitish-yellow on anterior half and yellow on posterior half except narrow portion along posterior margin somewhat darkened medially; head spots obscure but consisting of positive and negative spots: anteromedial and posteromedial spots and posterior spots of mediolateral spots faintly positive, and anterior spots of mediolateral spots and posterolateral spots faintly negative. Lateral surface of head capsule yellow except eye-spot region whitish, and area between eye-spot region and posterior margin somewhat darkened. Ventral surface of head capsule (Fig. 6a) yellow except elongate spot and posterior portion of each side of postgenal cleft darkened. Antenna composed of three segments and apical sensillum, slightly longer than stem of labral fan; proportional lengths of first, second, and third segments 1.00: 1.15–1.19: 0.65–0.73. Labral fan with 40 or 41 primary rays. Mandible (Fig. 6b) with mandibular serrations composed of two teeth; major and longer tooth at obtuse angle to mandible on apical side; comb-teeth decreasing in length from first to third. Hypostoma (Fig. 6c) with nine anterior teeth, median and corner teeth most prominent; lateral margins moderately serrate apically; six hypostomal bristles lying divergent posteriorly from lateral border on each side. Postgenal cleft (Fig. 6a) large, rounded, deep, 3.0–3.3 times length of postgenal bridge. Thoracic cuticle almost bare. Abdominal cuticle almost bare except each side of anal sclerite moderately covered with short colorless setae, many of which are stout and needle-like, and lateral bulges of last segment moderately covered with fine colorless setae. Rectal scales present. Rectal organ of compound lobes, each with 18–21 finger-like secondary lobules. Anal sclerite X-shaped, with broadened anterior arms 0.7 times length of posterior ones. Last abdominal segment bulged laterally but lacking ventral papillae. Posterior circlet with 84–90 rows of hooklets with up to 15 hooklets per row.

### Type-material

HOLOTYPE: Female, reared from a pupa collected from a small stream (width 0.2 m, depth 2–3 cm, water temperature 16.4 °C, shaded, altitude 1728 m, 06°01.397’N, 116°32.611’E), slow flowing in a natural forest, Mt. Kinabalu, Sabah, 18-VI-2014, by M. Sofian-Azirun, Z. Ya’cob, C.D. Chen & K.W. Lau. PARATYPES: Three males (form A), one male (form B), all reared from pupae, and one pupal exuviae, and seven mature larvae, same data as holotype; one female, one male (form A), both reared from pupae, and one pharate female, collected from a small stream (width 3.5 m, depth10-12 cm, water temperature 17.5 °C, shaded, altitude 1591 m, 06°00.665’N, 116°32.399’E), moderate to fast flowing in a natural forest, Mt. Kinabalu, Sabah, 18-VI-2014, by M. Sofian-Azirun, Z. Ya’cob, C.D. Chen & K.W. Lau; one male (form A), reared from pupa collected from a small stream (width 6.0 m, depth 0.5 m, water temperature 17.0 °C, partially shaded, altitude 1714 m, 06°01.337’N, 116°36.420’E), moderate to fast flowing in a natural forest, Mesilau, Kundasang, Sabah, 18-VI-2014, by M. Sofian-Azirun, Z. Ya’cob, C.D. Chen & K.W. Lau; eight females, three males (form A), six males (form B), two intersexes (one with female head, though frons much narrower, female abdomen, male wings, male legs, and thorax of intermediate sex with indefinite scutal color pattern; the other with female head, though frons much narrower, female wings, male legs, male abdomen except female terminalia with abnormal structures between ovipositor valves and paraprocts, and thorax of intermediate sex with indefinite scutal color pattern), all reared from pupae collected from a main channel of a stream (width 3.0-4.0 m, stream bed rocky, water temperature 23.0 °C, partially shaded), moderately flowing in a pasture, Bakalalan, Sarawak, Malaysia (altitude 928 m, 03°50.624’N, 115°36.312’E, 21-VIII-2008), by H. Takaoka.

### Depository of type-specimens

The holotype and paratypes of the new species are deposited in the Institute of Biological Sciences, Faculty of Science, University of Malaya, Kuala Lumpur, Malaysia.

### Ecological notes

The pupae of this species were collected attached to plastic sheets and dead leaves of trees in the water. Associated species were *S*. (*N*.) *borneoense* Takaoka, *S*. (*S*.) *beludense* Takaoka, *S*. (*S*.) *laterale* and *S.* (*S.*) *keningauense* Takaoka.

### Distribution

Borneo (Sabah, Sarawak and Kalimantan).

### Etymology

The species name *mirum* refers to the extraordinary finding that this new species has dimorphic males. The Latin word ‘mirus’ means ‘extraordinary’.

### ZooBank registration

To comply with the regulations set out in article 8.5 of the amended 2012 version of the International Code of Zoological Nomenclature (ICZN), details of the new species have been submitted to ZooBank. The Life Science Identifier (LSID) of the article is urn:lsid:zoobank.org:pub:27FDFE51-721B-488C-BAAF-010822CDD626. The LSID for the new name *Simulium mirum* is urn:lsid:zoobank.org:act:E8B9D049-317B-4F8F-9235-53222BC3A172.

## Discussion

This new species is striking in that there are two forms of males, which are reared from pupae with apparently the same external morphology: form A with a scutal pattern as in Fig. 1b and Fig. 4b linked with a smaller number of upper-eye facets in 12–14 vertical columns and 15 or 16 horizontal rows, and form B with a scutal pattern as in Fig. 1c and Fig. 4c linked with a larger number of upper-eye facets in 16 or 17 vertical columns and 17 or 18 horizontal rows. Beside these two different characters, both forms of males have the same morphological characters including the genitalia. The pupae of these two forms of males are morphologically indistinguishable from each other, and usually coexist in the same habitats, strongly suggesting a single species with dimorphism in the male scutal color pattern linked with a different number of upper-eye facets. Our DNA analyses show that both forms of males are conspecific, although they can be genetically differentiated by locations, a common observation in black flies [22, 23]. Further, our genetic data clearly show that *S. mirum* n. sp. is distinguished from four known members of the *S. melanopus* species-group.

In the family Simuliidae, genetically-inherited dimorphism in one sex, like the present new species, has not been known though sexual dimorphism is common. *Simulium* (*Notolepria*) *gonzalezi* Vargas & diaz Najera from Belize, Ecuador, Guatemala and Mexico was reported to show dimorphism in the males in that some males are dichoptic with a narrow frons and more elongated antennal segments similar to those of the female, whereas most males are holoptic [24–26]. Since dichoptic heads with eyes medially separated by a frons are a typical feminine expression (e.g., Fig. 1a), all or some of these males of *S.* (*N.*) *gonzalezi* with dichoptic heads might be sexual mosaics, a phenomenon (mainly caused by mermithid infections [27]) occasionally observed in various groups of black flies.

The cause of the dimorphism shown in the males of *S. mirum* n. sp. is unknown. Unlike the case of *S. gonzalezi,* there seems to be the least possibility, if any, that it has been caused by the intersexuality. It is because the heads of both form A and form B males are holoptic (a typical expression of the male heads, i.e., the left and right eyes medially contiguous, not separated by the frons) (Fig. 1b, c), though the numbers of the enlarged upper-eye facets are different between form A and form B, thus having nothing to do with the expression of the feminine head morphology. In general, the abnormalities resulted from the intersexuality are mostly expressed longitudinally (e.g., the head feminine, the thorax and abdomen masculine, or vice versa), or asymmetrically in paired features (e.g., the left side of the body feminine, the right side masculine, or vice versa), or intermediate (e.g., two intersex specimens in the type-material).

In species of the *S. melanopus* species-group, the male scutal color pattern is usually monomorphic but rarely polymorphic, as shown in Figure 183A-E of *S. dumogaense* Takaoka & Roberts from Sulawesi [21]. However, unlike this new species, no linkage of these different scutal color patterns with the number of upper-eye facets or other characters has been reported.

Dimorphism in the males of *S. mirum* n. sp. may have originated before this new species had spread in Sabah and Sarawak because it was observed in at least two locations (Timpohon and Mesilau) in Sabah and three locations (Bakalalan, Bario and Pueh) in Sarawak (HT, unpublished data). Further studies are needed to determine the frequency of occurrence of each male form in each population and the underlying chromosomal and genetic mechanisms of this phenomenon.

By the unique scutal color pattern (Fig. 1b and Fig. 4b), form A males of this new species are distinguished from six of eight species from Sabah and Sarawak, for which the males are known, i.e., *S. crassimanum*, *S. lardizabalae* Takaoka & Sofian-Azirun, *S. laterale*, and *S. murudense* Takaoka, Ya’cob & Sofian-Azirun, *S. nigripilosum* and *S. timpohonense* Takaoka & Sofian-Azirun. Form B males are similar to those of *S. timpohonense* in having the yellow fore coxa and ordinary scutal color pattern (Fig. 1c and Fig. 4c), but are distinguished from the latter species by the entirely darkened hind tibia.

The female of *S. mirum* n. sp. is similar to those of *S. maklarini* and *S. cheedhangi* Takaoka, Ya’cob & Sofian-Azirun in having the yellow fore coxa, but is distinguished from them by the haired basal portion of the radial vein and entirely darkened hind tibia.

The pupa of this new species is similar to that of *S. maklarini* in having a similar arrangement of the gill filaments and similar shoe-shaped cocoon but it is distinguished by having the medial area surrounding the facial trichomes moderately covered with tubercles (Fig. 5a) and by sharply edged annular ridges of the gill filaments (Fig. 5i) (c.f., tubercles are absent on the face, and the annular ridges of the gill filaments are not sharply edged in *S. maklarini* [28]).

The mature larva of this new species is distinguished from *S. laterale* by the shorter body length 4.5–5.0 mm and posterior circlet with 84–90 rows of hooks (c.f., the body length 6–7 mm, and the posterior circlet with 115–170 rows of hooks in the latter species), and from *S. cheedhangi* Takaoka, Ya’cob & Sofian-Azirun by the length ratio of the second antennal segment against the first (1.15–1.19) (c.f., 0.95–0.98 in *S. cheedhangi*). The larvae of five other related species are unknown.

## Conclusions

Based on DNA analyses, *Simulium* sp. is proven to show dimorphism in the male scutal color patterns linked with different numbers of upper-eye facets. This species is described as a new species. This is the first report of such a novel species with dimorphic males among the family Simuliidae.

## Ethics statement

All experiments were performed in accordance with relevant guidelines and regulations of the University of Malaya. The research protocols were regulated and approved by the University of Malaya. Prior to the commencement of the sample collections, permission was approved by The Board of Trustees of Sabah Parks, Malaysia (Reference Number: TS/PTD/5/4 Jld. 49:52). This study did not involve endangered or protected species.

## Additional file

Additional file 1:
**Figure S1.** Maximum likelihood phylogenetic tree of the *Simulium melanopus* species-group from East Malaysia based on COI gene. Bootstrap and posterior probability values [ML/MP/NJ/BI] are shown on the branches. **Figure S2.** Maximum likelihood phylogenetic tree of the *Simulium melanopus* species-group from East Malaysia based on COII gene. Bootstrap and posterior probability values [ML/MP/NJ/BI] are shown on the branches. **Figure S3.** Maximum likelihood phylogenetic tree of the *Simulium melanopus* species-group from East Malaysia based on 12S rRNA gene. Bootstrap and posterior probability values [ML/MP/NJ/BI] are shown on the branches. **Figure S4.** Maximum likelihood phylogenetic tree of the *Simulium melanopus* species-group from East Malaysia based on 16S rRNA gene. Bootstrap and posterior probability values [ML/MP/NJ/BI] are shown on the branches. (PDF 4278 kb)
