# Supplementary material for: Dimorphic male scutal patterns and upper-eye facets of Simulium mirum n. sp. (Diptera: Simuliidae) from Malaysia
Source: Parasit Vectors. 2016 Mar 9;9:136. doi: 10.1186/s13071-016-1393-9 (PMC4784304; doi:10.1186/s13071-016-1393-9)
Supplement: Additional file 1: — Figure S1. Maximum likelihood phylogenetic tree of the Simulium melanopus species-group from East Malaysia based on COI gene. Bootstrap and posterior probability values [ML/MP/NJ/BI] are shown on the branches. Figure S2. Maximum likelihood phylogenetic tree of the Simulium melanopus species-group from East Malaysia based on COII gene. Bootstrap and posterior probability values [ML/MP/NJ/BI] are shown on the branches. Figure S3. Maximum likelihood phylogenetic tree of the Simulium melanopus species-group from East Malaysia based on 12S rRNA gene. Bootstrap and posterior probability values [ML/MP/NJ/BI] are shown on the branches. Figure S4. Maximum likelihood phylogenetic tree of the Simulium melanopus species-group from East Malaysia based on 16S rRNA gene. Bootstrap and posterior probability values [ML/MP/NJ/BI] are shown on the branches. (PDF 4278 kb) [file 13071_2016_1393_MOESM1_ESM.pdf]

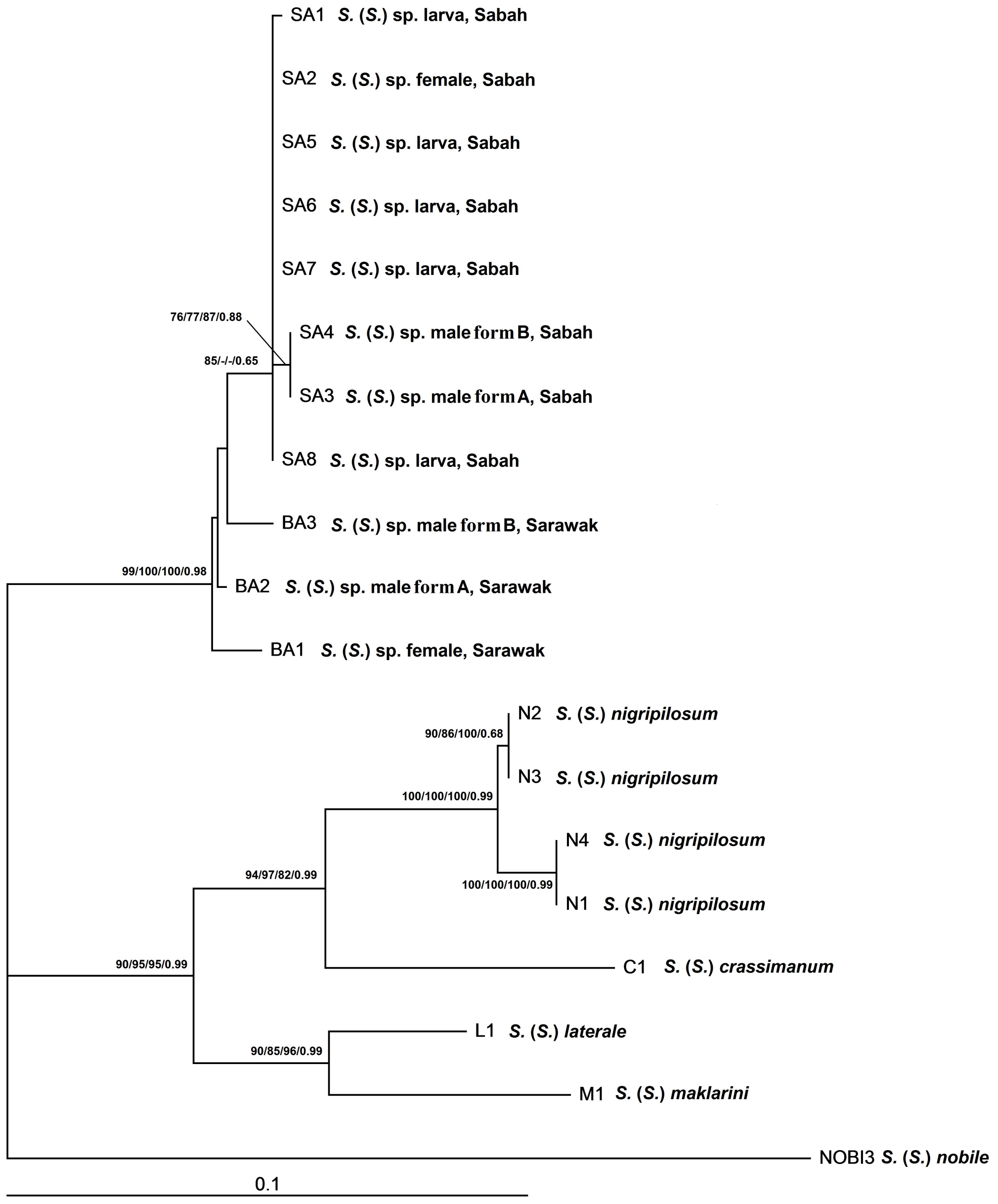

Figure S1 Maximum likelihood phylogenetic tree of the *Simulium melanopus* species-group from East Malaysia based on COI gene. Bootstrap and posterior probability values [ML/MP/NJ/BI] are shown on the branches.

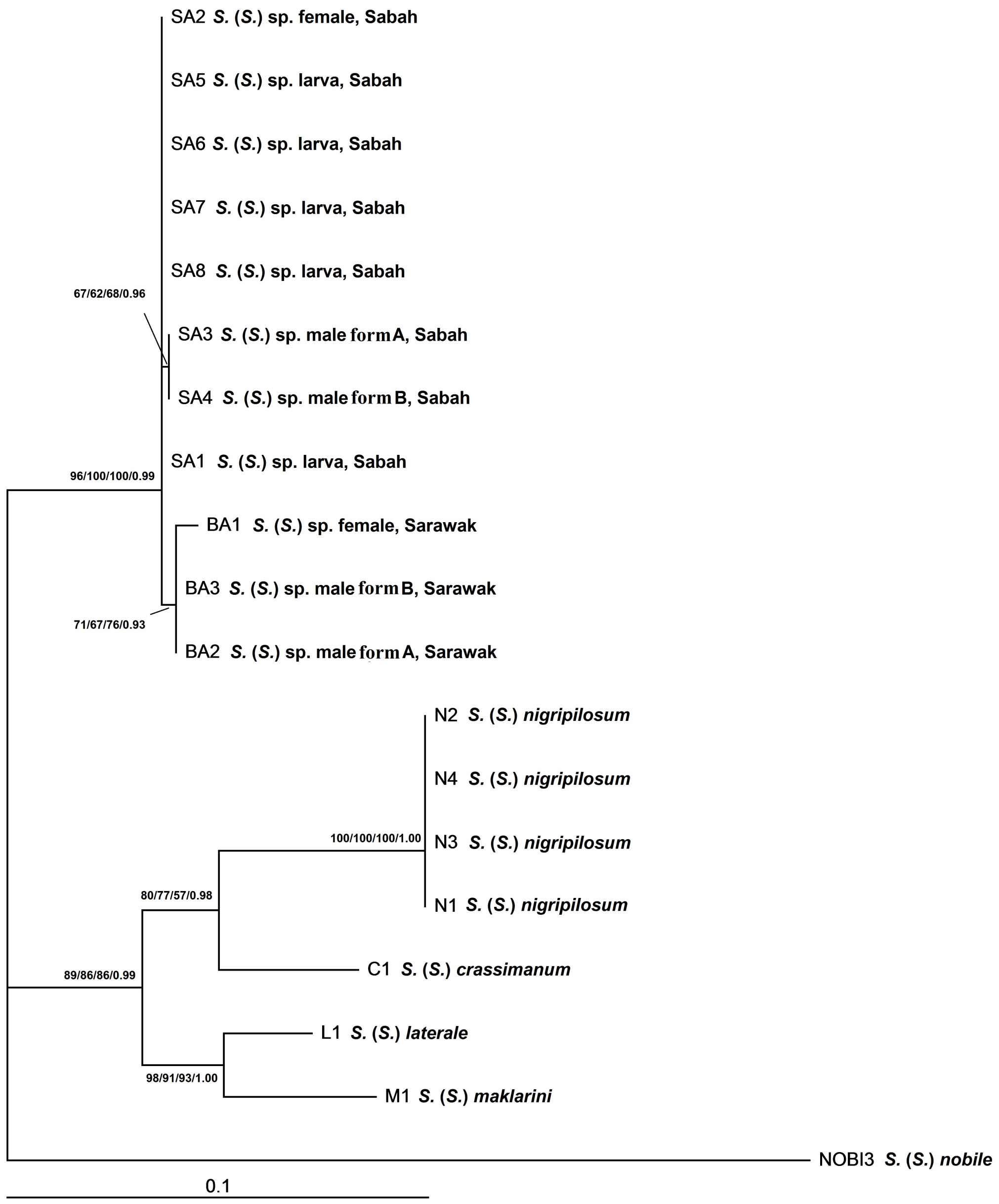

Figure S2 Maximum likelihood phylogenetic tree of the *Simulium melanopus* species-group from East Malaysia based on COII gene. Bootstrap and posterior probability values [ML/MP/NJ/BI] are shown on the branches.

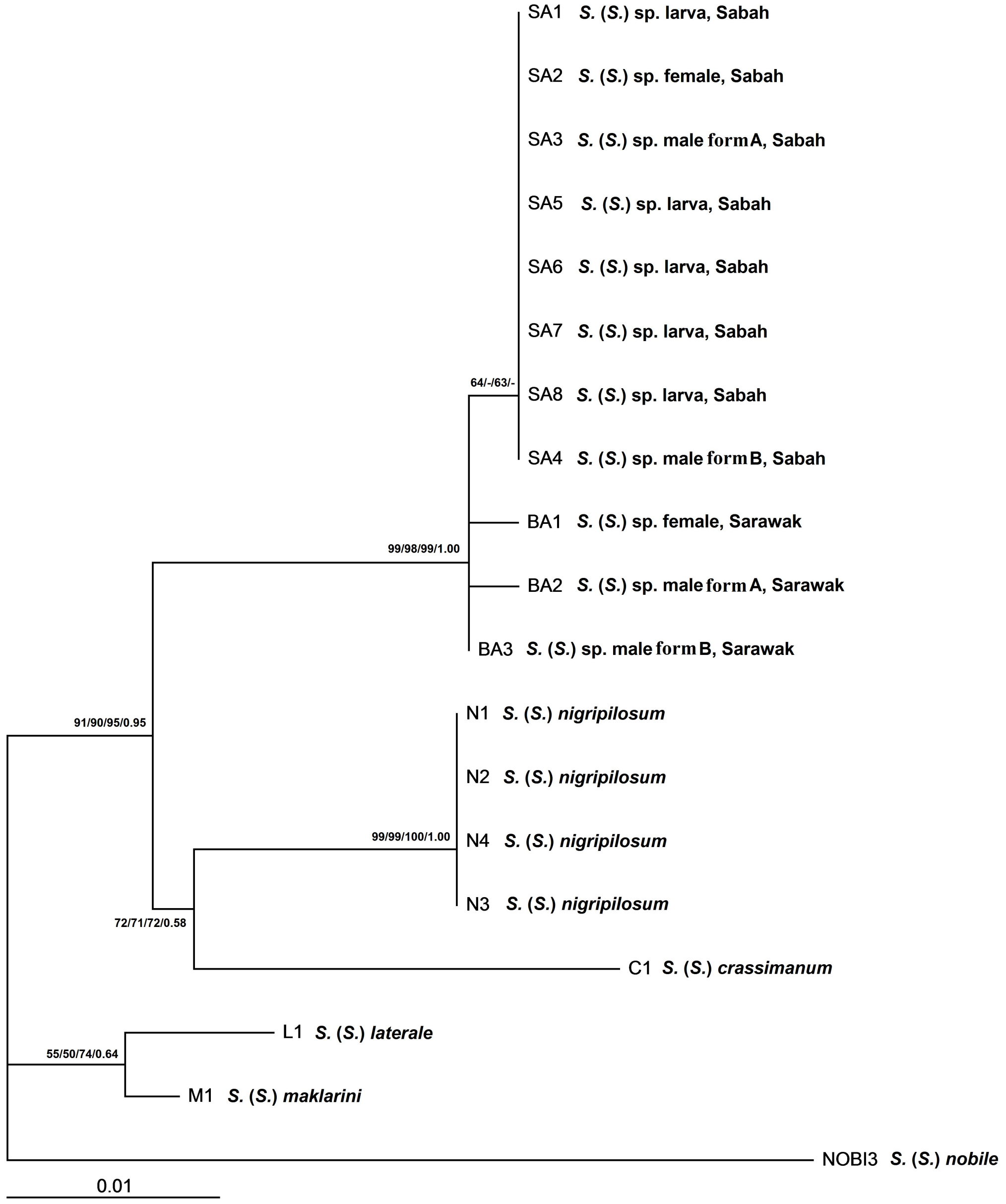

Figure S3 Maximum likelihood phylogenetic tree of the *Simulium melanopus* species-group from East Malaysia based on 12S rRNA gene. Bootstrap and posterior probability values [ML/MP/NJ/BI] are shown on the branches.

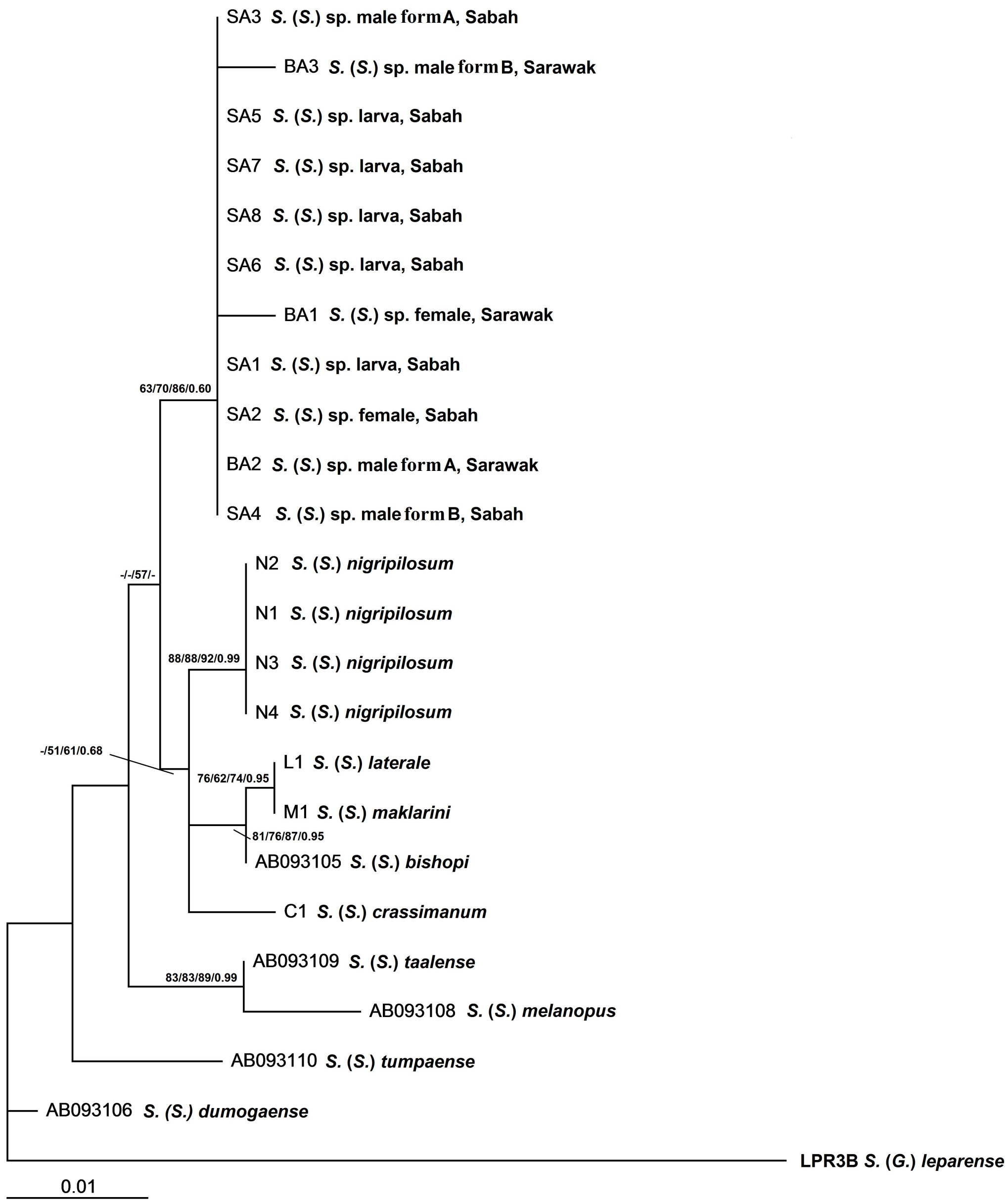

Figure S4 Maximum likelihood phylogenetic tree of the *Simulium melanopus* species-group from East Malaysia based on 16S rRNA gene. Bootstrap and posterior probability values [ML/MP/NJ/BI] are shown on the branches.
